# Immune Protection of Stem Cell-Derived Islet Cell Therapy for Treating Diabetes

**DOI:** 10.3389/fendo.2021.716625

**Published:** 2021-08-10

**Authors:** Meghan Tahbaz, Eiji Yoshihara

**Affiliations:** ^1^Lundquist Institute for Biomedical Innovation at Harbor-UCLA Medical Center, Torrance, CA, United States; ^2^David Geffen School of Medicine at University of California, Los Angeles, CA, United States

**Keywords:** diabetes, stem cells, human islet-like organoids, immune evasion, transcriptional memory

## Abstract

Insulin injection is currently the main therapy for type 1 diabetes (T1D) or late stage of severe type 2 diabetes (T2D). Human pancreatic islet transplantation confers a significant improvement in glycemic control and prevents life-threatening severe hypoglycemia in T1D patients. However, the shortage of cadaveric human islets limits their therapeutic potential. In addition, chronic immunosuppression, which is required to avoid rejection of transplanted islets, is associated with severe complications, such as an increased risk of malignancies and infections. Thus, there is a significant need for novel approaches to the large-scale generation of functional human islets protected from autoimmune rejection in order to ensure durable graft acceptance without immunosuppression. An important step in addressing this need is to strengthen our understanding of transplant immune tolerance mechanisms for both graft rejection and autoimmune rejection. Engineering of functional human pancreatic islets that can avoid attacks from host immune cells would provide an alternative safe resource for transplantation therapy. Human pluripotent stem cells (hPSCs) offer a potentially limitless supply of cells because of their self-renewal ability and pluripotency. Therefore, studying immune tolerance induction in hPSC-derived human pancreatic islets will directly contribute toward the goal of generating a functional cure for insulin-dependent diabetes. In this review, we will discuss the current progress in the immune protection of stem cell-derived islet cell therapy for treating diabetes.

## Introduction

Diabetes is a complex disease that affects more than 30 million people in the US alone and over 463 million people worldwide. Individuals with diabetes are subject to an increased risk for mortality due to cancers, infectious diseases, and other complications (1–6). The current COVID-19 pandemic has additionally highlighted that diabetes is a major risk factor for severe bouts of disease (7–18). In particular, type 1 diabetes (T1D) is a significant burden that typically appears during adolescence and requires life-long insulin administration and blood glucose monitoring (19). T1D is a chronic disease characterized by the autoimmune destruction of pancreatic islet β cells (20). This irreversible loss of insulin-producing β cells impairs crucial glucose uptake in peripheral tissues, resulting in hyperglycemia and subsequent life-threatening microvascular and macrovascular complications (21). Though the etiology of T1D remains unclear, it is known that both environmental risk factors and genetic susceptibility contribute to disease pathogenesis (22). The multifactorial nature of the disease precludes the discovery of a cure. However, the increasing global T1D prevalence, as well as the significant economic and social burdens, demands a solution (23). Currently, T1D and late-stage type 2 diabetes (T2D), a condition triggered by severe peripheral insulin resistance and β cell dysfunction, comes in the form of exogenous insulin administration. However, this method of insulin delivery is not an accurate substitute for normal pancreatic islet function, largely due to the lack of precise temporal glucose control (24). In comparison, allogeneic pancreatic islet transplantation offers a minimally invasive treatment option for T1D patients, which significantly improves glycemic control while preventing severe hypoglycemia (25). Although clinical trials indicate that pancreatic islet transplantation is a promising β cell replacement therapy (26, 27), there are two key issues that prevent its widespread therapeutic utilization. The first issue is a chronic shortage of human islets; the shortage of cadaveric pancreatic islets and the low yield of pancreatic islet purification directly limits transplantation use, whose efficacy depends on the number of functional islets that survive engraftment (28). Additionally, the cumulative cost of islet transplantation is exceedingly high, amounting to more than $120,000 in 2009 per surgical treatment (29), limiting the use of this therapy to only wealthy patients in high-income countries. The second issue is the immune rejection and loss of function of transplanted grafts; patients who have undergone pancreatic islet transplantation require immunosuppressive agents, such as thymoglobulin and basiliximab, to avoid graft rejection of transplanted islets (30–32). Chronic immunosuppression can lead to increased risks of infection, autoreactivity, and the induction of malignancies (33–35).

The shortage of human cadaveric islets, the high cost of islet transplantation, and the complications associated with life-long immunosuppression collectively demonstrate the crucial need for novel approaches to pancreatic islet transplantation. One promising avenue of research is the generation of functional pancreatic islets from human pluripotent stem cells (hPSCs) such as embryonic pluripotent stem cells (ESCs) (36) and induced pluripotent stem cells (iPSCs) (37), which possess indefinite self-renewal and pluripotency.

Chemically defined stepwise differentiation by using small molecules and recombinant proteins enables us to induce glucose-responsive, functional insulin-producing cells from hPSCs in a reproducible manner. hPSC-derived pancreatic islet β cells hold great promise as an alternative source of primary islets for treating diabetes, although there remain major limitations in maturity and graft survival due to immune rejection. *In vivo*, β cells become functionally mature *via* a long-term of postnatal maturation process. This process has not yet been duplicated *in vitro* to transform hiPSCs into fully functional β cells equivalent to primary β cells, indicating that further improvements in the hPSC-derived β cell maturation process are required. In addition, even though islet cells delivered from hiPSCs are by definition autologous (and thus MHC-matched), life-long immune suppression may still be required to protect against autoimmune rejection of transplanted islet cells due to a hyperactive immune reaction in T1D patients.

In this review, we will outline the current progress in generating functional human pancreatic islets as well as novel approaches to protect stem-cell derived islets from immune rejection. Lastly, we will discuss limitations that presently hinder the utilization of hPSC-derived β cells as a common diabetes therapy.

## Generation of Functional Human Islets From hPSCs

**Figure 1:**
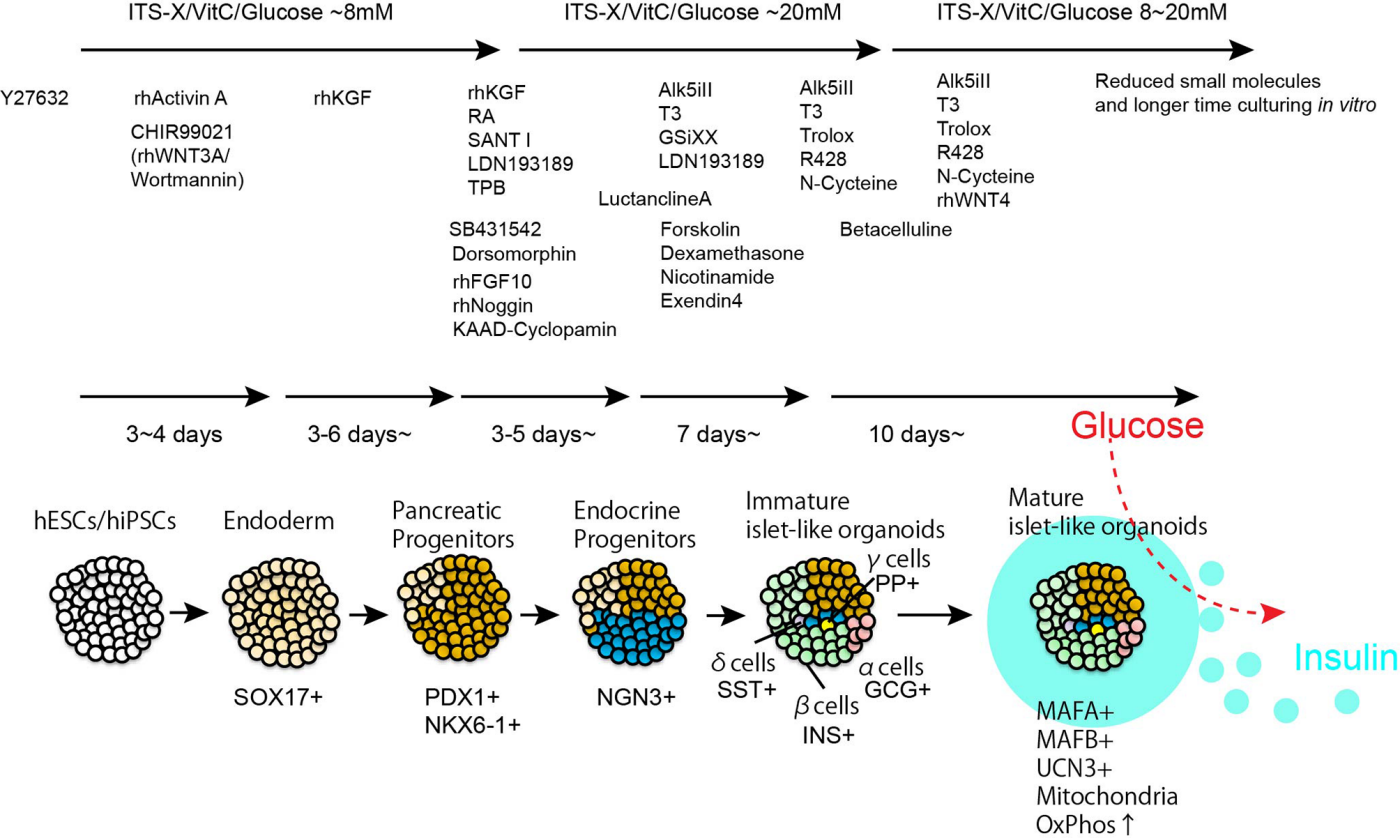
Step by step differentiation and maturation to generate functional human islet-like organoids. By using chemically defined recombinant proteins and small molecules, hPSCs are introduced pancreatic lineage specification. Islet-like organoids contains insulin producing β-cells, glucagon producing α-cells, somatostatin producing δ-cells, and pancreatic polypeptide producing γ-cells. The representative duration and small compounds/recombinant proteins used for β-cells differentiation are listed in the Figure.

Stem cell biology is a rapidly developing field with immense implications for regenerative medicine, including the treatment of diabetes. hPSCs successfully differentiate *in vitro* into pancreatic progenitors (PPs) and, more recently β-like cells, *via* differentiation protocols that rely on developmental paradigms and the introduction of small molecules that regulate stage-specific pathways (38) (**Figure 1**).

In 2006, D’Amour et al. developed a five-stage differentiation protocol capable of producing endocrine hormone–expressing cells from hESCs which is a step forward from their protocol for hESC-derived definitive endoderm differentiation (39); however, these insulin-expressing cells largely resemble immature fetal β cells in their glucose responsiveness, despite their promising insulin content (40). Differentiation efficacy has been improved from a few percent to over 10% by identifying significant pathways that regulate human pancreas development such as FGFs, WNTs, BMP, PKC and TGFβ signaling (41–47). However, several key differences between primary adult human β cells and stem cell-derived human β cells have been observed, such as aberrant populations of polyhormonal cells (48), functionality (49), transcriptome (50) and epigenetic profiling (51). In 2014, Pagliuca et al. and Rezania et al. reported a protocol to generate functional glucose-responsive human β cells (52, 53); a simplified protocol has also been reported (54). Notably, these insulin-producing cells reverse diabetes *in vivo* after transplantation into rodents (52–54). Although these pioneering protocols recaptured well-differentiated mono-hormonal β cell features, such as co-expression of PDX1, NKX6-1 and insulin/c-peptide and high glucose-stimulated insulin secretion, cells exhibited slow insulin secretion speeds in response to glucose and aberrant Ca^2+^ signaling, which are critical for insulin exocytosis (52, 53). The maturity of β cells can be characterized by two separate components. The first component of maturity is β cell lineage specification: how β cells express specific functional genes such as INS, IAPP, UCN3, MAFA, MAFB, G6PC2 and SIX2, and coordinate the proper β cell identity network (55–63). MAFA regulates INS and G6PC2 gene expression, which are required for enhancing insulin production, and suppressing insulin secretion at lower glucose concentrations to enhance the amplitude of glucose-stimulated insulin secretion (GSIS), respectively (64–66). Another component of β cell maturity is physiological metabolic regulation: how β cells regulate physiological metabolic activity which links to the amplification of GSIS. β cells exhibit unique features of glucose metabolism (67, 68). High expression of glucose transporters, such as GLUT1 in humans and Glut2 in rodents, rapidly equalizes extra- and intracellular glucose concentrations. Low glucose affinity glucokinase (GK) expression, instead of hexokinase (HK) expression, and low lactate dehydorogenese (LDH) and monocarbohydorate transporter (MCT) expression accumulates intracellular pyruvate, which is the primary fuel source of the citric acid cycle and mitochondrial oxidative phosphorylation to produce ATP (67, 69). The increased ATP/ADP ratio by glucose-stimulated fuel metabolic utilities triggers Ca^2+^ influx and insulin secretion. Fetal and neonatal immature β cells show poor glucose responsiveness and transition to postnatal functional maturation with enhanced mitochondrial metabolic function (70–80). Previously, we have identified a critical role for the nuclear receptor estrogen related receptor gamma (ERRγ) in the postnatal maturation of β cells (77). Coincident with weaning, neonatal β cells become glucose-responsive, acquiring the ability to secrete insulin in response to a high glucose challenge. We have previously reported that the postnatal increase in ERRγ expression orchestrates the metabolic maturation of β cells. Indeed, mice lacking ERRγ in β cells are glucose intolerant and fail to appropriately secrete insulin in response to a high glucose challenge (77). Furthermore, forced ERRγ expression in hiPSC-derived β-like cells (ERRγ-β-like cells) enhances the capacity for oxidative phosphorylation, resulting in significantly improved GSIS function in human β-like cells, both *in vitro* and *in vivo* (77). These findings support the notion that a metabolic maturation step driven by ERRγ provides the energetic capacity necessary for GSIS in hiPSC-derived β-like cells. Acknowledging the importance of 3-dimensional (3D) organization and cell-cell communications in organ function and the terminal differentiation of organ-specific cell types (81, 82), we also adapted our technology to generate 3D structured human pancreatic islet tissues called human islet-like organoids (HILOs) from pluripotent stem cells (83). HILOs similar in size to human islets (100-500 μm) have been generated in 5 weeks with newly identified maturation factors such as non-canonical Wnt family member 4 (WNT4), which enhances mitochondrial function to enhance GSIS function. Nair et al. have found that facilitating endocrine cell clustering *in vitro* promotes human stem cell-derived β cell maturation as demonstrated by increased mitochondrial oxidative respiration and robust insulin secretion (84). Additionally, circadian regulation (85), aberrant expression of glycolytic enzymes (86), and transforming growth factor β (TGF-β) modulation (87) during hPSC differentiation into β cells have been reported as key components in achieving GSIS with dynamic insulin secretion. Recent advances in the single-cell transcriptome approach have enabled the fine tuning of genomic maps in human islet maturation, while further studies to identify the effects of peripheral nerve innervation (88), mesenchymal stromal cell contributions (89) and other microenvironmental cues in human islet maturation are required to overcome the current limitations regarding the efficacy of differentiation and maturation of stem cell-derived islets. Instead of fully functional primary islets or functionally mature stem cell derived islets, pancreatic progenitors from hPSCs have been acknowledged as an alternative source for islet cell therapy. In 2008, Kroon et al. showed that hESC-derived PPs undergo further differentiation and maturation *in vivo* a few months post-transplantation (90), suggesting that transplantation of hPSCs derived PPs can recapitulate functional organogenesis *in vivo.* The idea to use hPSC derived PPs for transplantation to ameliorate T1D became popular and it remains open for discussion whether hPSC derived PPs or fully mature β cells are the better source for transplantation (91). A clinical trial using hPSC derived PP for treating diabetes is ongoing (ClinicalTrials.gov: NCT03163511, NCT02239354). Although Chromatin remodeling (51) and systemic gene regulation (92) provides the mechanistic insights, the particular cues which lead to *in vivo* functional maturation of hPSC derived PP has not been elucidated yet. Sex differences (93) as well as species differences (94) in functional maturation have been observed, suggesting that the efficacy of hPSC derived PP for treating preexisting diabetes may rely on the condition of patients. Advances in the generation of more functionally matured hPSC derived β cell enriched clusters or islets may offer less variation in efficacy for diabetic patients. However, the effort for generating fully mature bona fide human islets has not been ended yet. A better grasp on the complex mechanism of postnatal maturation, include unveiling precise collaboration of transcriptional factors (95) is crucial to optimize *in vitro* differentiation protocols.

## Immune Protection by Immunosuppressants and Macro/Micro Encapsulation Device

Pancreatic islet transplants are subject to both alloimmune and autoimmune reactivities, which can cause graft rejection or the progressive loss of islet function. To combat graft rejection, previous immunosuppression regimens for allogeneic islet recipients consisted of an induction phase targeting T lymphocytes, followed by the delivery of immunosuppressive maintenance agents such as calcineurin inhibitors, DNA antimetabolites, and corticosteroids (96). Although these drugs bolstered pancreatic islet allogeneic graft survival, they also displayed diabetogenic effects and direct toxicity toward pancreatic β cells (96–101). Moreover, whole body immune suppression increases the risk of cancer development, infectious diseases and other complications, limiting the enthusiasm for islet cell therapy (102). The publication of the Edmonton Protocol in 2000 illustrated that islet transplantation from multiple donors accompanied by a glucocorticoid-free immunosuppressive regimen could achieve graft survival and insulin independence in patients with T1D (103). This steroid-free protocol included an IL-2 receptor antagonist (daclizumab), sirolimus, and low-dose tacrolimus (103). However, researchers at the University of Chicago who assessed the short- and long-term outcomes of the Edmonton protocol concluded that although the patients remained insulin-free for five or more years after the initial transplantation, they suffered recurrent side effects as a result of immunosuppression (104). Recent strategies for immunosuppression have shown clinical improvements derived from modifications made to the Edmonton protocol. Hering et al. employed antithymocyte globulin, daclizumab, and etanercept as an induction protocols, along with a mycophenolate mofetil, sirolimus, and no or low-dose tacrolimus maintenance regimen (30). All eight study participants became insulin- independent, with five out of the eight achieving insulin independence for over a year; importantly, none of the study participants experienced immunosuppression-related adverse events (30). A follow-up phase 3 clinical trial examining human islet transplantation in T1D patients utilized a similar induction immunosuppression regimen of antithymocyte globulin (eventually replaced by basiliximab) and etanercept in addition to sirolimus and low-dose tacrolimus maintenance agents (31). At the 1-year mark, 87.5% of patients achieved an HbA_1c_ < 53 mmol/mol, with HbA_1c_ > 50 mmol/mol indicating diabetes (31, 105). An alternate immunosuppression regimen combined Fc receptor non-binding humanized anti-CD3 monoclonal antibody hOKT3γ 1 (Ala-Ala) and sirolimus induction agents followed by sirolimus and reduced‐dose tacrolimus for maintenance immunosuppression, resulted in a prolonged reduction in CD4+ T-cell expression in islet transplant recipients (106). Bellin et al. illustrated the efficacy of the anti-CD3 monoclonal antibody alone, as well as antithymocyte globulin combined with tumor necrosis factor-alpha (TNF-α) inhibition, over the previously used interleukin-2 receptor antibodies (IL-2RAb) (107). Several studies showed that an effective calcineurin inhibitor-free immunosuppression protocol using the co-stimulation blocker belatacept, achieved insulin independence in transplanted patients (108, 109). Kim et al. found that replacement of tacrolimus with the JAK3 inhibitor tofacitinib effectively suppresses immune responses in diabetic monkeys transplanted with MHC-mismatched allogeneic islets (110). All these efforts contribute to our understanding of immunosuppression strategies to avoid acute immune rejection of transplanted islets, although chronic rejection still remains a major issue (111). Therefore, modern studies on islet transplantation have focused on the development of protective encapsulation biomaterials and immune tolerance induction, particularly with regard to stem cell-derived β cells to overcome both the shortage of functional islet supply and immune rejection.

Many studies have examined the efficacy of transplanting encapsulated pancreatic islets to treat or cure T1D. Cell encapsulation in biomaterials with low immunogenic profiles directly protects grafts from rejection by the host immune system. Encapsulation systems such as the TheraCyte macroencapsulation device system, enable protection of the grafts from host immune cell infiltration *via* a physical barrier (112–116). A gas exchangeable enhanced O2 supply device has been developed to aim for enhancing the graft survival (117–119). This macroencapsulation device system is being utilized in an ongoing clinical trial of stem cell-derived pancreatic progenitor cells or primary human islets in T1D patients (116, 118). Alternatively, the naturally derived hydrogel alginate is the most common biomaterial employed in microencapsulation (120–129). When combined with divalent ions at physiological concentrations, alginate develops into a gel matrix suitable for cellular encapsulation. This biomaterial provides a selectively permeable membrane, which allows for the diffusion of oxygen, nutrients, and insulin as well as protects transplanted islets from intrinsic immune cell infiltration. This technology was first employed over 40 years ago and demonstrated that implanted alginate-microencapsulated islets could ameliorate diabetes for 2 to 3 weeks in a streptozotocin-induced diabetic rat model (120). In 1994, Soon-Shiong et al. reported that the intraperitoneal injection of alginate-microencapsulated human islets with low-dose cyclosporine normalized blood glucose over 9 months in a patient with T1D without affecting the function of a previously transplanted kidney (123). However, the capsules are prone to destruction by the foreign body response, which leads to fibrosis and loss of islet function, and therefore short-lived glycemic correction (122, 125, 128, 130, 131). Innovations in encapsulation technology have enabled major improvements in transplant function longevity. Altering the spherical dimensions of capsules, reducing transplant volume, and chemically modifying alginate biomaterials have improved biocompatibility and sustained graft function for long-term periods (124, 126, 132–135). Incorporating stem cell technology, Vegas et al. demonstrated that implanting hPSC-derived β cells encapsulated with triazole-thiomorpholine dioxide alginate corrects diabetes in immunocompetent mice, without any immunosuppression (134). The implanted islets exhibited appropriate glucose responsiveness and, upon removal at 174 days, still contained functional β cells (134). Bochenek et al. showed that chemically modified alginate derivatives reduce foreign body immune responses and improve graft survival and GSIS in allogeneic islets of non-human primates (NHP) for 4 months without the need for immunosuppression (135). Alagpulinsa et al. reported that CXCL12-containing sodium alginate-encapsulated hPSC-derived β cells transplanted into immunocompetent mice enhance insulin secretion, normalize hyperglycemia, and remain fully functional for more than 150 days without immunosuppression (136). The preliminary success of hPSC-derived β cell encapsulation and transplantation into diabetic mouse models and NHP provides a proof-of concept for treating T1D in humans using the same strategy. Additionally, in some cases of optimized pancreatic islet encapsulation technology, continuous immunosuppression in transplant recipients, including patients with T1D, is unnecessary (126, 134, 137–139). Further refinement of O_2_ and nutritional supplies with or without functional vascularization for long-term stability, as well as inquiries into human auto- and alloreactivity toward these alginate compositions, including newly developed nanofiber technologies (140, 141) will be necessary steps toward future clinical significance.

## Transplant Preconditioning

Transplant preconditioning has been investigated to improve islet transplantation outcomes. Early graft failure in pancreatic islet transplant recipients led to inquiries into the complex mechanisms underlying this process. While T-cell recognition of mismatched human leukocyte antigens (HLA) is a major cause of allogeneic graft rejection in T1D patients, hypoxia and inhospitable transplant microenvironments are additional barriers to transplant success. A key trigger of early graft failure, islet embolism during intraportal pancreatic islet transplantation is known to cause liver ischemia and subsequent apoptosis of transplanted pancreatic β cells *via* induction of pro-inflammatory cytokines (142). Paradoxically, exposing the liver to ischemic conditions prior to transplantation was found to significantly reduce early graft failure in mice by decreasing the mRNA levels of the pro-inflammatory cytokine interleukin-1β (IL-1β) as well as tumor necrosis factor-α (TNF-α) and by increasing the plasma levels of Interleukin-10 (IL-10), an anti-inflammatory cytokine (142–146). This stress-induced resistance induction may not only be beneficial by direct exposure, but also beneficial by indirect exposure to co-cultured cells. Hypoxia-preconditioned mesenchymal stem cells or primary hepatocytes were shown to significantly reduce reactive oxygen species (ROS) production and induce pro-apoptotic proteins, such as Bcl-2, to improve islet survival when co-cultured with human islets (147, 148). Alternatively, pancreatic islets can be directly preconditioned *via* strategic drug delivery. Rat and human pancreatic islets preconditioned with diazoxide, a potassium channel activator, and subsequently exposed to hypoxic conditions are protected from hypoxia-induced necrosis by an unknown mechanism and ameliorate hyperglycemia in STZ-induced diabetic rats and immune deficient mice, respectively (149). Transplanted mouse pancreatic islet allografts preconditioned with mitomycin c, an antitumor antibiotic, experience a median survival duration of 28 days without immunosuppression compared to 13 days in the control group (150). In an *in vitro* model, mitomycin c treatment suppressed allograft proinflammatory cytokine expression, decreased inflammatory cell infiltration, and upregulation of CD4+-suppressing regulatory T cells (150). A green tea polyphenol and anti-inflammatory agent, epigallocatechin 3-gallate (EGCG), also improves pancreatic islet transplant viability (151). Mouse pancreatic islets cultured in 100 µM EGCG-containing medium and transplanted under the kidney capsules of STZ-induced diabetic mice demonstrated preserved insulin secretion and decreased ROS production as a function of the Nrf2 pathway (151). Taken together, these findings indicate multiple avenues for further studies focusing on pancreatic islet protection against inflammatory immune responses, hypoxia, and the transplant microenvironment. Additionally, regulatory T-cell (Treg) therapies offer a personalized approach to transplant immune tolerance. Tregs play an important role in preventing autoimmunity, while their antigen specificity ensures that tumor and pathogen immunosurveillance is not hampered (152). After identifying and isolating graft-specific patient Tregs, these cells have been cultured *in vitro* to generate a clinically useful population (152). An alternate therapy utilizes chimeric antigen receptor (CAR) T-cells, patient-derived T-cells engineered to express a synthetic receptor targeted against a specific antigen (153). CAR-T cells also develop into Tregs with graft-specific antigen receptors (154). Importantly they can be utilized to combat HLA mismatches in transplant graft recipients. Since HLA-A2 population expression is significant and develops a substantial number of HLA-A2-mismatched transplants, HLA-A2-specific CAR-T cells have been developed in a mouse transplant model, which has successfully prevented HLA-A2+ T-cell-initiated graft versus host disease (155). However, challenges remain with CAR-T cell-related toxicity, which must be addressed before application in human transplant cases (156, 157). Nevertheless, these preconditioning approaches may be promising in the context of hPSC-derived β cells, whose culturing methodology can be adapted to optimize graft survival.

## Personalized Medicine

The emergence of hiPSC technology, which is derived from various somatic cells, has popularized the idea of personalized treatments in regenerative medicine. One such idea is autologous transplantation by using patients’ self-derived hiPSCs. There are several immunological barriers for transplant technologies, particularly in patients with T1D who intrinsically possess hyperactive immune responses targeted against pancreatic β cells. However, because hiPSCs can be derived from adult tissues, they unlock a powerful form of personalized medicine: fibroblast cells taken from patients, converted into hiPSCs, and differentiated into pancreatic islet β cells could be transplanted autologously. This practice reduces the risk of graft versus host disease by accounting for immune identity (158, 159). Unfortunately, autologous hiPSC production is expensive and time-consuming. To attempt to overcome these disadvantages, there has been the generation of a bank of stringently selected HLA-homozygous hiPSC lines, which can be differentiated and transplanted to a broad group of patients (160–163). Using this strategy, healthy donors with homozygous HLA-A, HLA-B, and HLA-DR have been selected and, according to the frequent HLA haplotypes in the population, hiPSCs have been selectively generated and preserved *via* cryopreservation (163). Although, this approach may be effective in certain countries with less diversity, it is anticipated that a large-scale bank will be needed to cover the entire global population. Moreover, even with autologous delivery strategies, transplanted hiPSC-derived insulin-producing cells would eventually be rejected due to the hyperactive immune reaction in which activated T-cells are presented insulin as antigens. This autoimmunity is, in some cases, connected to the HLA haplotypes HLA-DR4-DQ8 and HLA-DR3-DQ2, which have been identified as two major genetic risk factors for T1D development (21). Naturally, introducing pancreatic islets with mismatched HLA to transplant recipients compounds this innate immune response by activating T-cells (111). Therefore, in addition to MHC matching strategies, protecting transplanted insulin-producing cells from hyperactive T-cells in T1D may be required. Ultimately, the development of a universal hPSC population that evades immune detection is a major goal of T1D translational research.

## Engineering Stem Cell Derived Islet-Organoids for Immune Evasion

Generating universal hPSCs that resist both allogenic and autoimmune rejection would constitute a clear advance in regenerative medicine. The self-renewal function of hPSCs provides a superior opportunity for genomic modification *in vitro*. Genome engineering technologies such as TALEN (164, 165) or CRISPR (166–171)-based genetic modification offer more flexible HLA design and immune evasive function in both hESCs and hiPSCs. HLA-A and HLA-B deletion reduces antigen presentation, while sustaining HLA-C or HLA-E suppresses NK cell lysis, which reduces allogeneic responses in hPSCs (172–174). In addition to MHC matching, enhancing the ability of transplanted β cells to evade immune detection could be an alternative strategy for reducing the risk of autoimmune rejection. The major question is how β-cells can attain immune tolerance and thus evade autoimmune rejection by T-cells. Anecdotally, one T1D patient who successfully survived for 50 years with intensive insulin injection therapy still retained at least some functional glucose-responsive β-cells, which were surrounded by T-cells because of the presence of antigenic insulin but retained their function (175). Although the limited number of these cells was insufficient to rescue this T1D patient, the mechanism through which these β-cells evade T cell-mediated immune rejection remain unknown. β-cells manifest slow turnover, undergo limited proliferation, and thus lack regenerative capacity (176–178). Cancer cells evade T-cell recognition and autoimmune rejection by expressing the immune checkpoint molecule programmed death 1 ligand 1 (PD-L1/CD274), the ligand of the T-cell inhibitory receptor programmed death 1 (PD-1/CD273) ligand (179). Similarly, a correlation between immune tolerance and PD-1/PD-L1 has been observed in diabetic rodents (180). PD-L1 is expressed in pancreatic islet cells, where it suppresses self-antigen-reactive CD8+ T cells (181–183). *In vitro* PD-L1 expression in human islet cells and EndoC-βH1 cells is upregulated in response to stimulation with the interferon-γ (IFN-γ), and interferon-α (IFN-α) stimulation (184, 185). Interestingly, when islets isolated from PD-L1-deficient C57BL/6j mice are transplanted into STZ-induced diabetic BALB/c mice, there is increased allograft rejection, inflammatory cell tissue infiltration, and T cell alloreactivity (186). It was also shown that PD-L1 + murine β cells have also been found during immune tolerance in murine β cells in the non-obese spontaneous type 1 diabetic (NOD) mice model (180), however they evidently dedifferentiated and, hence, were non-functional. We have previously shown that forced PD-L1 expression significantly reduced immune cell infiltration into transplanted HILOs, ameliorated diabetes in a xenograft, and humanized allogenic immunocompetent environment for more than ~50 days, significantly longer than their PD-L1-lacking counterparts (83). Notably, PD-L1-expressing grafts contain less CD45+ immune cells, such as T-cells (83). Similarly, it has been shown that PD-L1 overexpression by a CRISPR knock-in system enabled partial protection from autoreactive T-cells modeled with CD19-expressing stem cell-derived functional human β like cells with CAR-T cells (187). Using an alternate approach, pancreatic islet grafts have been engineered with chimeric PD-L1/streptavidin oligomers (SA-PD-L1) (188, 189). More than 90% of these SA-PD-L1 grafts have been retained for over a 100-day observation period after transplantation into STZ-induced diabetic C57BL/6 mice undergoing a 15-day rapamycin course as transient immune suppression (188). Immune cell phenotyping has revealed that SA-PD-L1 induces peri-islet infiltration of FOXP3+ regulatory T-cells, serving as a practical local immune modulation to support long-term graft survival without chronic immune suppression (189). Thus, induction of PD-L1 and other immune-tolerance-aiding protein-induction presents an innovative approach to improving pancreatic islet graft longevity.

PD-L1 + β-cells possess immune tolerance in non-obese diabetic (NOD) mice (180), a common mouse model for T1D. However, these immune tolerant murine β-cells exhibit defective GSIS function, and even their β-cell identity is unclear (180). Our recent findings revealed that constitutive PD-L1 expression in HILOs rapidly ameliorates diabetes in an immune-competent environment (83). In healthy primary human islet β-cells, only a few β-cells express PD-L1. However, IFNγ induces PD-L1 expression in both cadaveric human islets and β-cells or non-β-cell clusters (GFP+ or -) in mature HILOs. However, in agreement with previous reports of cytokine-induced β-cell dysfunction (190–192), stimulation with >10 ng IFNγ for 24 hours significantly reduces gene expression governing GSIS function and β-cell identity such as INS-1, UCN3 and STY4 in HILOs. This suggests that IFNγ signaling paradoxically regulates β-cell dysfunction (defects in GSIS and β-cell identity gene expression) and protection (enhanced PD-L1 expression). Therefore, we hypothesized that short-term IFNγ stimulation is sufficient to induce PD-L1 expression while preserving β-cell function. Interestingly, short term (2 hours) exposure of low dose IFNγ (<10ng/ml) is sufficient to induce PD-L1 expression in HILOs, but the expression of PD-L1 was not sustained for more than 3 days (83). Innate immune mechanisms have evolved into a type of built-in memory (193). This memory is important for maintaining cellular adaptability, which in turn helps with preparedness for similar future events. For example, multiple LPS injections induce differential LPS responsiveness and modify the pathological features after stroke (194). Recent studies have also revealed that innate immune memory is induced not only immune cells but also other differentiated cells such as skin fibroblasts and neuronal cells to alter the transcriptional and physiological immune responses against second or third similar stimulation (194–196). To achieve stable PD-L1 expression without long-term IFNγ exposure, we hypothesized that multiple short-term (2 hours) IFNγ stimulation (MPS) causes transcriptional memory for sustainable PD-L1 expression, ideally without affecting functionality. We found that MPS by IFNγ induces sustainable PD-L1 expression in HILOs (MPS-HILOs) without adversely affecting insulin secretion (83). Furthermore, transcriptome analyses in MPS-HILOs revealed “*de novo*” anti-inflammatory gene induction by MPS (83). Indeed, MPS-HILOs showed resistance to IL1β-induced β cell dedifferentiation (83). We propose this phenomenon as a novel transcriptional memory system of β-cells for adaptive immune tolerance and evasion. Elucidating the specific mechanisms underlying transcriptional memory in pancreatic islets may provide a novel and effective strategy for inducing immune tolerance in transplant recipients. These findings raise the possibility of generating immune-tolerant mature functional human islets from hPSCs *in vitro*, which in turn may offer a novel therapeutic approach to avoid graft and autoimmune rejection without immune-suppressive drugs in T1D patients.

## Conclusion

**Figure 2:**
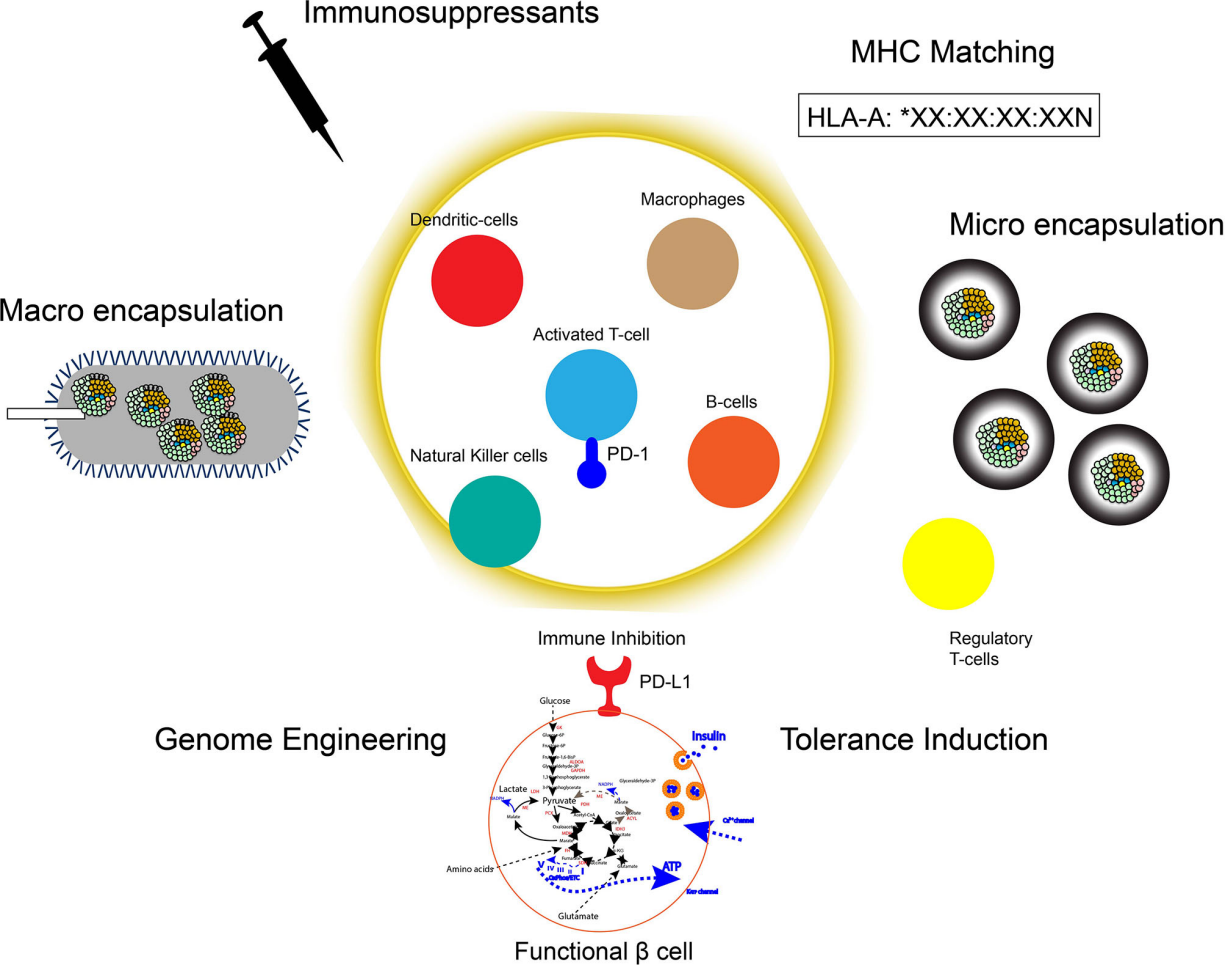
Strategies for immune protection of hPSC derived islets. To protect the transplanted hPSC derived islets graft: 1. Immunosuppressants, 2. Macro encapsulation devices (e.g. Theracyte Device), 3. Micro encapsulation gels (e.g. Alginate-coating), 4. MHC-matching, 5. Genome engineering of immune checkpoint molecules and/or HLAs, 6. Induction of immune tolerance by preconditioning or trained immunity.

Immune-evasive human islets derived from hPSCs represent a promising and renewable cell source with a reduced risk of chronic immune suppression to treat insulin-dependent diabetes. The manufacturing scalability of hPSC-derived β cells or islet organoids must also be addressed before its widespread clinical application can become a reality. Although, there has been success in generating α-like cells from hPSCs (197, 198), no protocol has been successful in generating each endocrine hormone cell independently. Understanding the fine-tuning lineage specification as well as the spatial information of intracellular communication in islet organoids may further improve the efficacy of differentiation and maturation in stem cell derived islets for clinical use. Additionally, improvements in immune protection, particularly with respect to encapsulation biomaterials, T cell or hPSC engineering, preconditioning, and immune tolerance induction, will strongly impact the long-term efficacy of islet cell therapy in both T1D and T2D (**Figure 2** and **Table 1**). Although creating immune evasive human islets is a major goal in achieving standardized islet cell therapy in diabetes, there are potential concerns to develop unwilling driverless cells, such as teratomas, with future events of infection or health complications. A suicide system represented by induced caspase 9 with chimeric dimers eliminates the hPSC-derived cells and may therefore be a potential safeguard system against this concern (199). However, the current suicide system induces approximately 95% apoptosis in hPSCs and hPSC-derived cells, suggesting that further improvements of the system to ensure the 100% elimination of transplanted cells are required (200). The challenges for the long-term survival of hPSC-derived immune evasive derived β cells or islet organoids with improved nutrition and oxygen supply as well as controlled graft microenvironment will make the safe harbor of stem-cell derived islets transplanted into patients with diabetes one step closer to a reality.

**Table 1:** Summary of immune protection studies for stem cell-derived islets or primary islets.

Strategy	Method		Target Cell Type	Side Effect(s)	Predicted Efficacy (Allo or Auto)	*In vivo* transplantation	Reference
**Gene targeting/Engineering**	PD-L1 overexpression		β-cells	Unknown	Allogeneic Autoimmune	Yes (Rodents)	(83) (187)
**Immune suppressants**	Induction immunosuppression (e.g. antithymocyte globulin, basiliximab, etanercept) with maintenance agents (e.g. sirolimus, tacrolimus)		T-cells, NKT cells, etc.	Induction of malignancies, greater risk of infection, autoreactivity	Allogeneic	Yes (Rodents)	(30) (31) (107–110)
					Autoimmune	Yes (Human)	
**MHC matching**	Generation of HLA-homozygous hiPSC lines		hiPSCs, hESCs	Unknown	Allogeneic	Yes (Rodents)	(172–174)
**Macro encapsulation**	Protection of grafts from host immune cell infiltration via a physical barrier	Theracyte device	β-cells	Fibrotic responses, Prevention of vascularization in the graft, Hypoxia	Allogeneic	Yes (Rodents)	(112–116)
		βAir device		Fibrotic responses, Prevention of vascularization in the graft	Autoimmune	Yes (Human)	(117, 118)
		Nanofiber		Prevention of vascularization in the graft, Hypoxia			(119, 140, 141)
**Micro encapsulation**	Encapsulation of grafts in biomaterials with low immunogenic profiles	Alginate	β-cells	Unknown	Allogeneic	Yes (Rodents)	(120, 121, 123–128, 130, 132–139)
		SA-PDL-1		Unknown	Autoimmune		(188, 189)
**Preconditioning**	Exposure of grafts to ischemia, hypoxia, or co-culturing to enhance immune tolerance and graft survival		β-cells or co-cultured cells (e.g. mesenchymal stem cells, primary hepatocytes)	Unknown	Allogeneic	Yes (Rodents)	(142, 143, 146–151)
**Transcriptional memory**	Multi-pulse IFNγ stimulation to induce transcriptional memory to induce PD-L1 expression and *De novo* cytokine tolerance		β-cells	Unknown	Allogeneic	Yes (Rodents)	(87)
					Autoimmune		

## Author Contributions

MT and EY wrote and edited the manuscript. EY conceptualized and obtained funding for this study. All authors contributed to the article and approved the submitted version.

## Funding

This work was supported by the funding from CTSI-UCLA awards, California Institute for Regenerative Medicine (CIRM)-DISC2 discovery award, Integrated Islet Distribution Program (IIDP) Pilot award, Allen foundation grant, Mishima Kaiun Memorial Foundation Research award and Lundquist Institute Voucher award.

## Conflict of Interest

EY is inventor on licensed patents and patent applications related to the HILOs technology described in this manuscript.

The remaining author declares that the research was conducted in the absence of any commercial or financial relationships that could be construed as a potential conflict of interest.

## Publisher’s Note

All claims expressed in this article are solely those of the authors and do not necessarily represent those of their affiliated organizations, or those of the publisher, the editors and the reviewers. Any product that may be evaluated in this article, or claim that may be made by its manufacturer, is not guaranteed or endorsed by the publisher.
